# An Essay on the Medical Properties of Sanguinaria Canadensis

**Published:** 1847-05

**Authors:** Isaac Thorn

**Affiliations:** Yellow Springs, Ohio; Clifton


Art. II.—*An Essay on the Medical Properties of Sanguinaria Canadensis.* By Isaac Thorn, M.D., of Yellow Springs, Ohio.

In view of the neglect with which our indigenous articles of the materia medica have too generally been treated, I have determined to lay before the profession my experience in regard to the therapeutic powers of the *Sanguinaria Canadensis*, a plant often mentioned by medical writers, but the virtues of which appear to be but imperfectly understood. In his elaborate and learned system of Materia Medica, Pereira refers to the blood-root as having been used with favorable results in diseases of the lungs and in rheumatism, and he also mentions that it has been given in jaundice, but the propriety of this practice he considers questionable. Dr. Eberle devotes three pages of his Materia Medica to the consideration of the medical properties of the plant, and quotes a number of authors who have found it a valuable therapeutic agent. Among others he refers to Dr. Francis, of New York, who reports having used it successfully in diseases of the chest, and also in a case of rheumatism. Dr. Eberle gives his own favorable experience in the use of the remedy, especially in cases of asthma; and he quotes Dr. McBride as having administered it with success in a case of hydrothorax. In the United States Dispensatory, by Wood and Bache, the sanguinaria is mentioned in terms of approbation. The authors of that work state that many judicious physicians have borne strong testimony to its curative powers; but, like the other writers quoted, they do not appear to have arrived at any very satisfactory conclusions as to what are its peculiar properties.

According to the analysis reported by Eberle, the sanguinaria contains cinchonine, extractive matter, resin, a gummy matter, and gallic acid in a state of combination. Dr. Dana, of New York, has found in it a peculiar principle to which he has given the name of *sanguinarina*, and which, no doubt, is the active principle of the root. It is described as a white pearly substance, of an acrid taste, which renders the yellow of tumeric brown, changes the infusion of purple cabbage green, unites with acids, and shows all the other qualities of an alkali.

Writers concur in regarding the sanguinaria as a stimulant, acrid emetic, and narcotic, to which alterative, tonic, and diaphoretic properties have been added by some; but the number of published observations needs to be extended before its *modus operandi* can be accurately determined.—During the last two years I have been closely engaged in observing the effects of the remedy in various diseases, and in this time have used a large quantity of it. The result of my trials with the root is, that it is a *sedative* of no ordinary powers. I should say that, for reducing the force and frequency of the pulse, at the same time without prostrating the system, it is one of our most efficient remedies. In this respect, I have found that it possesses very decided advantages over tartar emetic and digitalis. Its sedative effects are exerted slowly, not being perceived until after the medicine has been administered twenty-four to forty-eight hours, and then come on very gradually. Eberle notices this fact.

I have also found the sanguinaria to possess true *alterative* properties. Under its use the secretions of the liver are manifestly affected, and, with this change, the condition of the stomach improves. I have administered it in a variety of glandular obstructions, and its action in such cases has been highly satisfactory.

The sanguinaria unquestionably also exerts a *tonic* influence on the system, but this it does, I am disposed to think, by virtue of the action just referred to. Restoring as it does healthy secretion, the tone of the system will certainly improve under it. With the returning appetite and powers of digestion, the complexion rapidly improves, and this effect has been so striking, that I have been strongly inclined to believe, that there was something in the bloodroot which increased directly the quantity of blood corpuscles. Whether this conjecture has any foundation in fact or not, my observations have satisfied me that we possess few remedies which more efficiently aid the recuperative energies of the system than the sanguinaria. And in this connection I would refer to the experience of veterinary practitioners, which is not without its bearing upon this subject. In the disease of horses known by the name of *yellow water*, the puccoon root is esteemed by farriers the very best remedy. This disease is attended by extreme debility, emaciation, and an impoverished condition of the blood. The serum is largely out of proportion to the solid constituents; in other words, the blood exhibits all the characters found in it in anemic diseases, and the paleness of complexion and debility of the animal point to the same condition of things. Considering the resemblance of this affection in the horse to chlorosis in the human subject, I should expect benefit from the use of the blood-root in the latter complaint. I have not had an opportunity of making the trial, but it occurs to me as one worthy of being made, especially since the remedy would be likely to exert a beneficial influence aside from any changes that it might induce directly in the blood. But it will be more profitable to dismiss these conjectures for the present, and give some of the results at which I have arrived from my experiments with the remedy.

Case I.—Mr. A., æt. about 40 years, a respectable merchant of Clifton, after exposure to inclement weather, was attacked with pneumonia. I found him with high fever, cough, severe pain in the chest, and expectorating phlegm tinged with blood. He was treated by venesection, tartar emetic, and blisters, for two or three days, when all the symptoms of pneumonia suddenly disappeared, but were succeeded by those of dysentery. The pains in his bowels were severe; the abdomen became tympanitic, and all his symptoms assumed a threatening aspect. On consultation with Doct. Gillett, of Springfield, it was concluded to put him on an infusion of the sanguinaria in such doses as his stomach would bear. His pulse, at the time he began to take this remedy, ranged from 120 to 130, and was tense and corded. After a lapse of twenty-four hours the pulse began to improve, and in two days had declined to 80; and with the amendment in this respect all the other symptoms improved, and convalescence was soon established.

Case II.—The Rev. R. F. consulted me in the summer of 1845, respecting an inactive state of the liver, with which he had been troubled for several years. He was dyspeptic, had dullness of intellect, an icterode complexion, and suffered from constipation of the bowels. I put him upon sanguinaria combined with taraxacum, as follows:

℞. Sanguinar. pulv.
Tarax. ää ʒj.

M., divide into 30 pills, of which take two morning, noon, and night. Under their use the patient soon began to mend. His digestion was better, bowels became soluble, his head clear, and he was finally cured. I might detail other cases analogous to this, but content myself with reciting one of each affection in which I have employed the remedy.

Case III.—Mrs. C., of this neighborhood, aged about 35 years, consulted me in respect to a goitre of eight or ten years' standing. She was the mother of four children, of a slender form, lymphatic temperament and a scrofulous family, her mother having died of scrofula and several of her sisters exhibiting the taint. I prescribed tincture of sanguinaria, 20 drops, three times daily, conjoined with two grains of the iodide of iron. After a few weeks the latter was discontinued and the sanguinaria given alone, in order that the powers of this article might be fairly tested in the case. At the time the lady entered upon the treatment, the tumor was as large as a man's fist; in six months it was less than a hen's egg. During the use of the sanguinaria her menstrual flux became excessive, and, supposing that the root might have some connexion with this, she was directed to intermit the remedy for a time. The menses were less copious at the next period, and by the second or third they had become natural in quantity. She now resumed the use of the sanguinaria, and at the next return her catamenia was too copious; but her strength is not in the slightest degree affected by the profuse discharge, and in fact her general health has decidedly improved in the two years during which she has used the remedy. The tumor, though materially diminished in size, has not entirely disappeared.

In several cases I have witnessed the same effect upon the uterus from the use of the sanguinaria, and I was visited, this morning, by a lady just relieved by it of dysmenorrhœa.

Case IV.—Mrs. B., aged about 35 years, in the spring of 1835, consulted me on account of difficulty of breathing. This difficulty was increased by the recumbent posture, and was attended by cough, slight œdema of the lower extremities, and, in a word, the general symptoms of hydrothorax. She was at the time pregnant with her second child. I gave her cathartics and diuretics, of which calomel formed a constituent, and by this plan prevented the disease from advancing until after the birth of the child. Soon after that event the symptoms became aggravated, and, having tried a variety of remedies inefficiently, she was put upon the sanguinaria. One of the early and promising effects of the remedy was an improvement in her circulation; which was soon followed by the subsidence of the œdema and other hydropic symptoms.

She continued to mend under the use of the medicine and finally recovered her health.

Case V.—April 29th, 1846, I was called to Mrs. C., the patient of a neighboring practitioner. I found her sitting propped up in bed and breathing laboriously. I learned from the physician in attendance that, a fortnight before, she was seized with a pain in the left side, accompanied by fever, and that she had been bled and blistered for the affection. On examination, I discovered water in the left side. So urgent were the symptoms that, on consultation, it was decided to practice *paracentesis thoracis*, and accordingly I introduced a scalpel between the fifth and sixth ribs of the left side. More than a gallon of fluid was discharged which coagulated on cooling. Immediate relief to the respiratory apparatus followed the operation.

The patient was now put upon the following prescription: ℞. calomel one grain, and sanguinaria and squills of each, in powder, two grains, to be repeated every three hours. She persevered in the course for three days, when her stomach became so irritable that it was found necessary to discontinue the squills and calomel. The dose of sanguinaria was considerably increased, so as to bring the circulation under its influence. For about eight days the fluid in the chest appeared to be returning, but from that time forward there was no increase, and in a month it had disappeared, and the patient was able to be about her work. It is remarkable that, in this case also, something like menorrhagia supervened upon the use of the blood-root, and has persisted up to the present time, notwithstanding which the lady enjoys better health than she did before the attack of hydrothorax. She now has her catamenia upon her nearly three weeks out of every four.

In several cases of hemoptysis I have employed the sanguinaria with success. Females, it is known, frequently have a discharge of blood from the lungs which is vicarious of the menstrual flux. In cases of this character no article of the materia medica possesses greater efficacy than the blood-root.

I have used this remedy repeatedly in jaundice, and the results of my trials with it have been highly satisfactory. Its applicability to this disease, suggested at first by the color of the root, is now no longer questionable. In all cases of jaundice depending upon a functional disorder of the liver, I should place my reliance upon the sanguinaria.

In intermittent fever, some physicians of my acquaintance regard this article as nearly equal in efficacy to the preparations of cinchona. As a general remedy in this disease I should not be disposed to confide in it; but there is a form of intermittent fever to which it appears to me to be well adapted. I refer to that which appears in the spring after an autumnal attack, and which does not depend directly upon miasmatic poison, but upon derangement in the chylôpoietic viscera. In cases of this description I have used the sanguinaria with the happiest effects. In such cases it appears to me far superior to the preparations of bark.

This root ought not to be kept on hand too long. My impression is that after a year it loses much of its power, and I should therefore urge the employment of a fresh article.

I have used all the officinal preparations of sanguinaria, and have also given it in combination with iodine and iron, and several vegetable medicines. The compound of proto-iodide of iron and sanguinaria is one which is applicable to a variety of diseases, and is capable of fulfilling important indications. I have also found the combination of sanguinaria and taraxacum a good one.

The use of sanguinaria is contraindicated by a plethoric state of the system, by an inflammatory tendency, and by gastric and intestinal inflammation in the acute stage. In subacute inflammation of these organs I have used the remedy with success.

If this indigenous medicine should turn out as successfully in the hands of others as it has proved in mine, I venture nothing in predicting that the time is not distant when it will be esteemed one of the most excellent articles of the materia medica.

Clifton, March 9th, 1847.

